# Effect of cholesterol variability on the incidence of cataract, dementia, and osteoporosis: A study using a common data model

**DOI:** 10.1097/MD.0000000000035548

**Published:** 2023-10-13

**Authors:** Jong Sung Park, Do-Hoon Kim, Byong-Kyu Kim, Kyeong-Hyeon Park, Dong Ho Park, Yang Ha Hwang, Chang-Yeon Kim

**Affiliations:** a Department of Internal Medicine, Kyungpook National University Hospital, Daegu, Republic of Korea; b Division of Cardiology, Department of Internal Medicine, Severance Cardiovascular Hospital, Yonsei University College of Medicine, Seoul, Republic of Korea; c Medical Big Data Research Center, Kyungpook National University Hospital, Daegu, Republic of Korea; d Department of Nuclear Medicine, Daejeon Eulji Medical Center, Eulji University School of Medicine, Daejeon, Republic of Korea; e Division of Cardiology, Department of Internal Medicine, Dongguk University, College of Medicine, Gyeongju Hospital, Gyeongju, Republic of Korea; f Department of Orthopedic Surgery, Severance Children's Hospital, Yonsei University College of Medicine, Seoul, Republic of Korea; g Department of Ophthalmology, Kyungpook National University Hospital, School of Medicine, Kyungpook National University, Daegu, Republic of Korea; h Department of Neurology, Kyungpook National University Hospital, School of Medicine, Kyungpook National University, Daegu, Republic of Korea; i Department of Internal Medicine, Daegu Catholic University Medical Center, School of Medicine, Daegu Catholic University, Daegu, Republic of Korea.

## Abstract

The effects of cholesterol variability on cataracts, dementia, and osteoporosis remain controversial. Using a common data model, we investigated the effects of variations in cholesterol levels on the development of cataracts, dementia, and osteoporosis. Patients who received statin therapy between 2011 and 2020 and those with 3 or more tests for total cholesterol (TC), low-density lipoprotein cholesterol (LDL-C), high-density lipoprotein cholesterol (HDL-C), and triglyceride (TG) levels were included. The patients were divided into those with a coefficient of variation (CV) of TC higher than the mean (high-CV group) and those with a lower CV of TC (low-CV group). Moreover, 1:1 propensity score matching was conducted based on demographic variables. Cataract, dementia, or osteoporosis was defined as having a diagnostic, drug, or surgical code based on the cohort definition. Of the 12,882 patients, cataracts, dementia, and osteoporosis were developed in 525 (4.1%), 198 (1.5%), and 438 (3.4%) patients, respectively. The stratified Cox proportional hazards model showed that the incidences of cataracts and osteoporosis were 1.38 and 1.45 times greater in the high-CV group than in the low-CV group, respectively. Our study revealed that TC variability is associated with developing cataracts and osteoporosis.

## 1. Introduction

As the elderly population increases, interest in geriatric diseases is likewise increasing. Cataracts, dementia, and osteoporosis are the most prevalent degenerative diseases. Among them, cataract is one of the causes of visual impairment in older adults worldwide, contributing to the primary ophthalmological public health problem.^[1]^ The lens lipid composition is crucial to its structure, but the impact of serum cholesterol levels in patients with or without statin therapy on the onset of cataracts is debatable.^[2–4]^

Similarly, dementia and osteoporosis may be associated with serum cholesterol levels. Notably, cell biology and gene studies support the association between cholesterol and the development of Alzheimer disease.^[5]^ Additionally, cholesterol and cholesterol-lowering medications regulate bone metabolism.^[6]^ However, these effects are controversial and have yet to be demonstrated in real-world data. Few studies have focused on the association between fluctuations in serum cholesterol levels and the development of cataracts, dementia, and osteoporosis.

The common data model (CDM) was used to standardize medical records and data from multicenter collaborative studies. Owing to the availability of big clinical data and medical information on patient care, such as diagnosis, medication, surgery, and examinations, CDM was the ideal method for this study.^[7–11]^ Moreover, the personal information of the patients was anonymized. As the CDM is accessible in large-scale medical databases, uncommon complications of rare diseases can be identified.^[12]^ Furthermore, CDM is becoming increasingly popular in research in various medical disciplines owing to its benefits.

There is increasing interest in the influence of intra-individual variability and physiological measures on disease development. In the CDM measurement table, intra-individual variability can be obtained without difficulty and used in research. Therefore, this study aimed to determine the effect of serum cholesterol level variability on cataracts, dementia, and osteoporosis using the CDM.

## 2. Methods

### 2.1. Ethical approval

This study was approved by the Institutional Review Board of Kyungpook National University Hospital (KNUH IRB No.2023-01-008) and conducted in accordance with the Declaration of Helsinki (1989) by the World Medical Association.

### 2.2. Data source

This retrospective observational study was performed at Kyungpook National University Hospital. We used the Observational Health Data Sciences and Informatics open-source software and the Observational Medical Outcomes Partnership CDM version 5.3 database, into which the electronic medical record (EMR) data were transformed. The database contains de-identified EMR data formatted according to the industry standards of the OMOP CDM system. Over nearly 15 years, beginning in 2006, the EMR data of over 2 million patients were transformed into our hospital CDM database.

### 2.3. Registry-based patient selection

**Figure 1.**
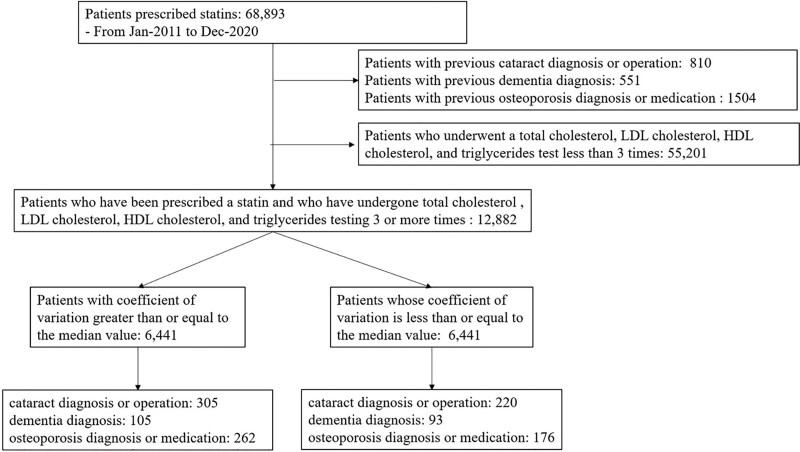
Flow diagram of the study.

In this retrospective registry-based case series study, we analyzed 68,893 patients from the CDM database who received statin therapy between January 2011 and December 2020. The cohort registry compiled patient demographic data. We excluded from the analysis the patients who had previously undergone cataract diagnosis or operation (n = 810), those who had previously undergone dementia diagnosis (n = 551), those who had previously undergone osteoporosis diagnosis or medication (n = 1504), and those who underwent TC, LDL-C, HDL-C, and TG tests less than 3 times (n = 55,201). Finally, 12,882 patients were included in this study (Fig. 1).

### 2.4. Variables

The mean, standard deviation, and coefficient of variation (CV) of the cholesterol test results were calculated for each patient. We excluded variables that could not be identified in the EMR or that were converted to the CDM. Moreover, we collected demographic factors (age and sex), comorbidities, and clinical data from the Centers for CDM and Prevention database. We identified comorbidities in the CDM database using SNOMED-CT. Laboratory measurements were recorded using concept IDs defined by the Logical Observation Identifier Names and Codes,^[13]^ an international standard for identifying medical laboratory observations (Supplementary Table 1, http://links.lww.com/MD/K201). Finally, patients with cataracts were defined as those with a diagnostic or surgical code for cataracts.

### 2.5. Data analyses

The patients were divided into those with a CV of total cholesterol (TC) higher than the mean (high-CV group) and those with a lower CV of TC (low-CV group). Cataract occurrence was confirmed by recording the diagnosis or operation, dementia by diagnosis, and osteoporosis by diagnosis or medication. To compare the 2 groups, 1:1 propensity score (PS) matching was conducted for sex, age, and comorbidities (hypertension, diabetes mellitus, osteoporosis, renal disease, rheumatoid arthritis, mild liver disease, heart failure, chronic pulmonary disease, anemia, mood disorder, previous sepsis, dementia, hypothyroidism, Parkinson disease, peripheral vascular disease, urinary tract infection, peptic ulcer disease, and cerebrovascular disease).

### 2.6. Statistical analysis

Statistical analysis was conducted using R software version 4.0.4 (R Foundation for Statistical Computing, Vienna, Austria). Variables are expressed as mean ± standard deviation, median (minimum-maximum), or number (frequency) where appropriate. Chi-square and Fisher exact tests were used to compare categorical variables. Similarly, *t* tests were used to compare the continuous variables.

A PS weighting method was applied to further minimize the effects of selection bias and potential confounding factors. Moreover, a logistic regression model was used to regress the treatment status on the observed baseline characteristics, and the estimated PS represented the predicted probability of treatment derived from the fitted regression model. Variables associated with study outcomes or clinical relevance were included and measured at baseline. Furthermore, the comparison was performed using PS matching by matching the subjects in a 1:1 ratio. The logit of the PS was often used as the matching scale, and the matching caliper was 0.2 × SD [logit (PS)]. After matching, all standardized mean differences were <0.1.

In univariate analyses, the Cox proportional hazards model was used to compare the hazard rates between the high CV and low-CV groups. A stratified Cox proportional hazards model was used for matched data. Hazard ratios (HRs) for cataract, dementia, and osteoporosis were compared between the high and low CV groups. The cumulative incidence of cataracts, dementia, or osteoporosis from the first statin therapy was estimated using the Kaplan–Meier method. Data pre-processing and analyses were performed using R version 4.0.4 (R Foundation for Statistical Computing, Vienna, Austria).

## 3. Results

### 3.1. Demographic characteristics

Of the 12,882 patients, 7677 (59.6%) were male. The mean age was 59.5 ± 11.9. Among them, cataracts, dementia, and osteoporosis developed in 525 (4.1 %), 198 (1.5 %), and 438 (3.4 %) patients, respectively. In the high-CV group, 305 (4.7 %), 105 (1.6 %), and 262 (4.1 %) patients had cataracts, dementia, and osteoporosis, respectively (Fig. 1). PS matching, statistically significant differences in age, hypertension, renal disease, mild liver disease, and the prevalence of cataracts and osteoporosis were observed between the high- and low-CV groups (Table 1). However, after PS matching, no statistically significant differences were observed in the comorbidity factors between the groups, except for the prevalence of cataracts and osteoporosis (Table 2).

**Table 1:** Patients’ demographic data.

	Low-CV group (n = 6641)	High-CV group (n = 6641)	*P* value	Standardized mean difference
Sex, n (%)			.473	0.013
Female	2582 (40.1)	2623 (40.7)		
Male	3859 (59.9)	3818 (59.3)		
Age (yr), mean ± SD*	60.39 ± 11.56	58.62 ± 12.17	<.001	0.15
Hypertension, n (%)*	1337 (20.8)	1055 (16.4)	<.001	0.113
Diabetes mellitus, n (%)	1048 (16.3)	1033 (16.0)	.738	0.006
Rheumatoid arthritis, n (%)	62 (1.0)	71 (1.1)	.486	0.014
Renal disease, n (%)*	308 (4.8)	446 (6.9)	<.001	0.091
Mild Liver disease, n (%)*	326 (5.1)	392 (6.1)	.013	0.045
Heart failure, n (%)	228 (3.5)	188 (2.9)	.052	0.035
Chronic pulmonary disease, n (%)	129 (2.0)	107 (1.7)	.168	0.025
Anemia, n (%)	74 (1.1)	67 (1.0)	.611	0.01
Mood disorder, n (%)	109 (1.7)	128 (2.0)	.238	0.022
Previous sepsis, n (%)	6 (0.1)	6 (0.1)	1	<0.001
Hypothyroidism, n (%)	284 (4.4)	294 (4.6)	.702	0.007
Parkinson disease, n (%)	21 (0.3)	11 (0.2)	.111	0.031
Peripheral vascular disease, n (%)	113 (1.8)	115 (1.8)	.947	0.002
Urinary tract infection, n (%)	19 (0.3)	30 (0.5)	.152	0.028
Peptic ulcer disease, n (%)	87 (1.4)	72 (1.1)	.264	0.021
Cerebrovascular disease, n (%)	520 (8.1)	494 (7.7)	.413	0.015

Data are expressed as mean ± SD or number (percent).

CV = coefficient of variation, high-CV group = patients with coefficient of variation of total cholesterol higher than the mean, low-CV group = patients with coefficient of variation of total cholesterol lower than the mean, SD = standard deviation.

* *P* value < .05.

**Table 2:** Comparison analysis by propensity score matching (1:1).

	Low CV group (n = 5920)	High CV group (n = 5920)	*P* value	Standardized mean difference
Sex, n (%)			.955	0.001
Female	2385 (40.3)	2389 (40.4)		
Male	3535 (59.7)	3531 (59.6)		
Age (yr), mean ± SD	59.74 ± 11.50	59.70 ± 11.55	.833	0.004
Hypertension, n (%)	1037 (17.5)	1044 (17.6)	.885	0.003
Diabetes mellitus, n (%)	918 (15.5)	946 (16.0)	.496	0.013
Rheumatoid arthritis, n (%)	58 (1.0)	57 (1.0)	1	0.002
Renal disease, n (%)	304 (5.1)	300 (5.1)	.900	0.003
Mild Liver disease, n (%)	315 (5.3)	313 (5.3)	.967	0.002
Heart failure, n (%)	187 (3.2)	183 (3.1)	.874	0.004
Chronic pulmonary disease, n (%)	104 (1.8)	104 (1.8)	1	<0.001
Anemia, n (%)	55 (0.9)	65 (1.1)	.409	0.017
Mood disorder, n (%)	103 (1.7)	111 (1.9)	.629	0.01
Previous sepsis, n (%)	6 (0.1)	6 (0.1)	1	<0.001
Hypothyroidism, n (%)	265 (4.5)	273 (4.6)	.757	0.006
Parkinson disease, n (%)	11 (0.2)	11 (0.2)	1	<0.001
Peripheral vascular disease, n (%)	101 (1.7)	102 (1.7)	1	0.001
Urinary tract infection, n (%)	19 (0.3)	23 (0.4)	.643	0.011
Peptic ulcer disease, n (%)	64 (1.1)	72 (1.2)	.546	0.013
Cerebrovascular disease, n (%)	445 (7.5)	463 (7.8)	.557	0.011

Data are expressed as mean ± SD or number (percent).

CV = coefficient of variation, high-CV group = patients with coefficient of variation of total cholesterol higher than the mean, low-CV group = patients with coefficient of variation of total cholesterol lower than the mean, SD = standard deviation.

### 3.2. Comparison of the low CV group and high CV group

Cox proportional hazards model analysis revealed 1.36 times (HR:1.36, 95% CI 1.27 to 1.45, *P* < .001) higher cataract occurrence and 1.42 times (HR:1.42, 95% CI 1.32–1.52, *P* < .001) higher osteoporosis occurrence in the high CV group than in the low CV group. Dementia occurrence was 1.11 times (OR:1.11, 95% CI 0.97–1.26, *P =* .446) greater in the high CV group than in the low CV group, but there was no statistically significant difference (Table 3). Stratified Cox proportional hazards model by matching 1:1 PS based on sex, age, and comorbidities showed 1.38 times (HR:1.38, 95% CI 1.28–1.47, *P* < .001) higher cataract occurrence and 1.45 times (HR:1.45, 95% CI 1.35–1.56, *P* < .001) higher osteoporosis occurrence in the high CV group than in the low CV group (Table 3).

**Table 3:** Comparison of outcomes between low-CV group with high-CV group.

Outcomes	Low CV group n = 6641	High CV group n = 6641	Univariate n = 12,882 Cox proportional hazards model		Matched n = 11,840 stratified Cox proportional hazards model	
	n (%)	n (%)	Hazard Ratio (95% CI)	*P* value	Hazard Ratio (95% CI)	*P* value
Cataract	220 (3.4)	305 (4.7)	1.36 (1.27–1.45)	<.001	1.38 (1.28–1.47)	<.001
Dementia	93 (1.4)	105 (1.6)	1.11 (0.97–1.26)	.446	1.34 (1.19–1.49)	.054
Osteoporosis	176 (2.7)	262 (4.1)	1.42 (1.32–1.52)	<.001	1.45 (1.35–1.56)	<.001

CI = confidence interval, CV = coefficient of variation, low-CV group = patients with coefficient of variation of total cholesterol lower than the mean, high-CV group = patients with coefficient of variation of total cholesterol higher than the mean.

**Figure 2.**
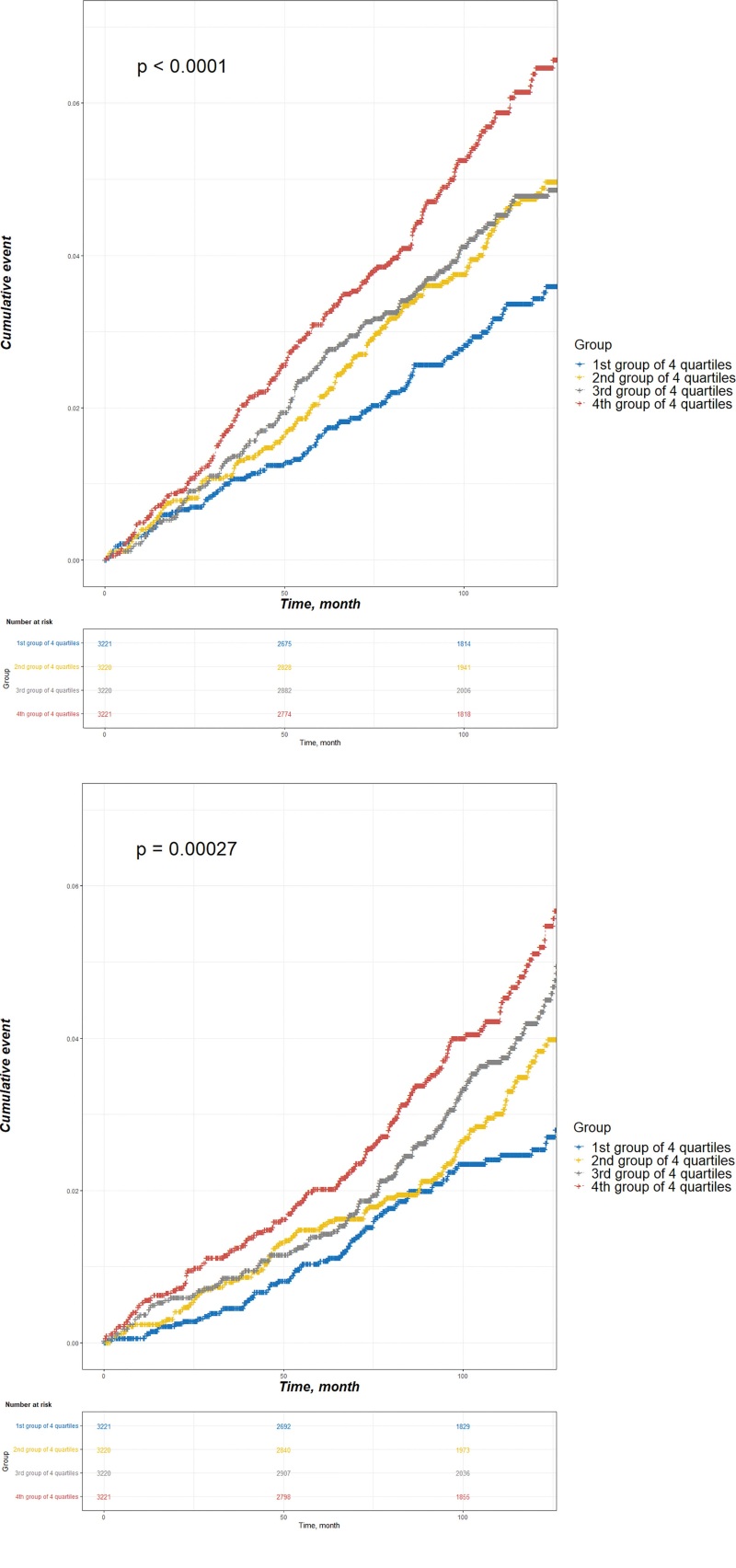
Survival curves of the cataract (A) and osteoporosis (B) according to quartiles of total cholesterol coefficient of variation.

Figure 2 illustrates the survival curves of cataracts and osteoporosis based on the variation in TC according to the CV quartile. Significant differences in cataracts and osteoporosis were observed among the 4 groups (*P* < .001). Compared with the group with minor variations, the group with substantial variations had a greater sequential incidence of cataracts and osteoporosis.

## 4. Discussion

Using a CDM database, this study discovered that individual TC variability was related to the development of cataracts and osteoporosis after accounting for confounding variables using PS-matched demographic and comorbidity variables after PS matching. However, no correlation was observed between TC variability and increased risk of dementia.

Notably, cholesterol variability is associated with certain diseases. Recent research has shown that various degenerative or cardiovascular disorders, including dementia, myocardial infarction, and stroke, are more likely to occur when TC levels vary considerably.^[14,15]^ Moreover, variations in each type of cholesterol are independently associated with developing certain diseases and major adverse cardiac events.^[16,17]^

Several studies have investigated the association between the occurrence of cataracts and cholesterol levels, with or without statin use. However, their findings were inconsistent.^[2–4]^ Therefore, the underlying mechanism of the association between lipid profile and cataracts needs to be clarified. Several hypotheses have been proposed. First, the components of metabolic syndrome, such as abdominal obesity, high blood pressure, and impaired fasting glucose, are well-known risk factors for cataracts.^[18]^ Specifically, individuals with metabolic syndrome are likely to have high cholesterol levels and fluctuations. Second, because the human lens contains cholesterol, variations in serum cholesterol levels may directly affect lens degeneration.^[19,20]^ However, further studies are required to investigate this mechanism. A similar effect of cholesterol has been observed in osteoporosis, in which high cholesterol levels and variation may be risk factors for osteoporosis by directly preventing osteoblast growth.^[21]^

In contrast, many studies have investigated the association between dementia and cholesterol levels.^[22,23]^ Lee et al reported that an increase in LDL-C level was associated with an increased risk of dementia among statin users.^[24]^ Moreover, Chung et al demonstrated that higher variability in TC was associated with an increased risk of all-cause dementia, independent of mean TC, in the general population.^[14]^ However, their findings were inconsistent with ours, probably because of the different study populations. Therefore, future studies involving various populations are warranted.

This study had some limitations. First, there may be confounding factors and biases due to this single-center, retrospective observational study. Despite the 1:1 PS matching analysis used to reduce bias, there might have been additional bias due to insufficient demographic and comorbidity variables. Future multicenter studies should compensate for these limitations. Second, a large number of patients who underwent TC, LDL-C, HDL-C, and TG tests less than 3 times were excluded, potentially resulting in a selection bias towards a relatively less healthy population. Third, we did not consider that differences in cholesterol variability could be a result of disease or disease intervention/treatment. Fourth, we did not consider the uncertainty with which CV was estimated. In the future, it would be useful to model repeated measures of cholesterol,^[25]^ ideally using joint models, to fully estimate the association between variability and disease outcomes.^[26]^ Fifth, aside from most demographic characteristics, there was no information on the risk factors for cataracts, dementia, or osteoporosis, which may be confounding factors. Finally, the study focused only on statin users, which may restrict the generalizability of the findings to other populations.

## 5. Conclusion

Our finding revealed that variability in TC was associated with the development of cataracts and osteoporosis among patients with statins. Further multicenter studies using the CDM should be conducted to validate these associations.

## Acknowledgments

We thank Jisun Kim from the department of medical informatics at Kyungpook National University Hospital for her data assistance in preparing this manuscript.

## Author contributions

**Data curation:** Do-Hoon Kim.

**Formal analysis:** Do-Hoon Kim.

**Methodology:** Do-Hoon Kim.

**Software:** Do-Hoon Kim.

**Supervision:** Byong-kyu Kim, Kyeong-Hyeon Park, Dong Ho Park, Yang Ha Hwang, Chang-Yeon Kim.

**Writing – original draft:** Jong Sung Park.

**Writing – review & editing:** Chang-Yeon Kim.

## Supplementary Material
