# Supplementary material for: Effect of cholesterol variability on the incidence of cataract, dementia, and osteoporosis: A study using a common data model
Source: Medicine (Baltimore). 2023 Oct 13;102(41):e35548. doi: 10.1097/MD.0000000000035548 (PMC10578724; doi:10.1097/MD.0000000000035548)
Supplement: Supplementary file 1 [file medi-102-e35548-s001.doc]

**Supplement Table 1. Concept identification for database: measurement, diagnosis, medication (lipid modifying agents, drugs affecting bone structure and mineralization), and procedure**

| Concept ID | Concept Name | Domain |
| --- | --- | --- |
| Cholesterol test | | |
| 4008265 | Total cholesterol measurement | Measurement |
| 4012479 | Low density lipoprotein cholesterol measurement | Measurement |
| 4032789 | Triglycerides measurement | Measurement |
| 4101713 | High density lipoprotein cholesterol measurement | Measurement |
| Cataract condition | | |
| 376400 | Combined form of senile cataract | Condition |
| 376401 | Traumatic cataract | Condition |
| 376973 | Cataract secondary to ocular disease | Condition |
| 381295 | Senile cataract | Condition |
| 432895 | Cortical senile cataract | Condition |
| 438749 | Posterior subcapsular polar senile cataract | Condition |
| 439297 | Nuclear senile cataract | Condition |
| 760555 | Bilateral age-related cataract | Condition |
| 764824 | Age-related nuclear cataract of right eye | Condition |
| 4001501 | Calcified cataract | Condition |
| 4008296 | Coronary cataract | Condition |
| 4102186 | Glaucoma in endocrine, nutritional and metabolic diseases | Condition |
| 4109548 | Drug-induced cataract | Condition |
| 4130588 | Morgagnian cataract | Condition |
| 4161420 | Juvenile cataract | Condition |
| 4220818 | Infantile cataract | Condition |
| 4230391 | Nuclear sclerotic cataract | Condition |
| 4230930 | Punctate cataract | Condition |
| 4301387 | Cataracta brunescens | Condition |
| 4317977 | Bilateral cataracts | Condition |
| 4336000 | Proliferative retinopathy with neovascularization elsewhere than the optic disc due to diabetes mellitus | Condition |
| 35624213 | Secondary cataract | Condition |
| 36684732 | Chronic iridocyclitis of left eye | Condition |
| 36684792 | Bilateral chronic anterior uveitis of eyes | Condition |
| 37108936 | After-cataract of bilateral eyes | Condition |
| 37208005 | Senile combined form cataract of right eye | Condition |
| 40479994 | Presenile cataract | Condition |
| 40482507 | Incipient senile cataract | Condition |
| 45757567 | Hypermature senile cataract | Condition |
| Dementia condition | | |
| 372608 | Amnestic disorder | Condition |
| 373179 | Senile degeneration of brain | Condition |
| 377254 | Multi-infarct dementia, uncomplicated | Condition |
| 378419 | Alzheimer's disease | Condition |
| 379778 | Multi-infarct dementia | Condition |
| 381270 | Parkinson's disease | Condition |
| 441947 | Posttraumatic state | Condition |
| 443605 | Vascular dementia | Condition |
| 443790 | Multi-infarct dementia with delusions | Condition |
| 443864 | Multi-infarct dementia with depression | Condition |
| 444091 | Multi-infarct dementia with delirium | Condition |
| 762696 | Fracture of odontoid process type I | Condition |
| 4043738 | Hydrocephalus | Condition |
| 4044416 | Post-traumatic mutism | Condition |
| 4046089 | Vascular dementia of acute onset | Condition |
| 4046090 | Mixed cortical and subcortical vascular dementia | Condition |
| 4047745 | Traumatic encephalopathy | Condition |
| 4047747 | Subcortical vascular dementia | Condition |
| 4048875 | Senile dementia | Condition |
| 4140090 | Parkinsonism | Condition |
| 4159643 | Senile dementia with psychosis | Condition |
| 4182210 | Dementia | Condition |
| 4190891 | Cerebral autosomal dominant arteriopathy with subcortical infarcts and leukoencephalopathy | Condition |
| 4218017 | Primary degenerative dementia of the Alzheimer type, presenile onset | Condition |
| 4220313 | Primary degenerative dementia of the Alzheimer type, senile onset | Condition |
| 4264860 | Specimen type not specified | Condition |
| 4295956 | Mood disorder due to a general medical condition | Condition |
| 36562408 | Bowen disease of other specified parts of female genital organs | Condition |
| 37111242 | Delirium co-occurrent with dementia | Condition |
| 37117216 | Delirium due to multiple etiological factors | Condition |
| 42538857 | Subcortical dementia | Condition |
| 43021816 | Mixed dementia | Condition |
| 43530664 | Dementia of the Alzheimer type with behavioral disturbance | Condition |
| 44782422 | Dementia due to Parkinson's disease | Condition |
| 45766122 | Cerebral autosomal recessive arteriopathy with subcortical infarcts and leukoencephalopathy | Condition |
| Osteoporosis condition | | |
| 77365 | Disuse osteoporosis | Condition |
| 77630 | Disorder of shoulder | Condition |
| 80502 | Osteoporosis | Condition |
| 80824 | Senile osteoporosis | Condition |
| 81390 | Idiopathic osteoporosis | Condition |
| 133570 | Acute osteomyelitis of pelvic region and/or thigh | Condition |
| 4002133 | Postoophorectomy osteoporosis | Condition |
| 4002134 | Osteoporosis in endocrine disorders | Condition |
| 4003481 | Vertebral osteoporosis | Condition |
| 4003482 | Postoophorectomy osteoporosis with pathological fracture | Condition |
| 4003483 | Osteoporosis of disuse with pathological fracture | Condition |
| 4004623 | Localized osteoporosis - Lequesne | Condition |
| 4010333 | Postmenopausal osteoporosis | Condition |
| 4019513 | Brittle diabetes mellitus | Condition |
| 4033089 | Drug-induced osteoporosis | Condition |
| 4057798 | Corrosion of ankle and foot | Condition |
| 4067765 | Drug-induced osteoporosis with pathological fracture | Condition |
| 4067768 | Postmenopausal osteoporosis with pathological fracture | Condition |
| 4069306 | Idiopathic osteoporosis with pathological fracture | Condition |
| 4069524 | Osteoporosis with pathological fracture of lumbar vertebrae | Condition |
| 4105090 | Regional osteoporosis | Condition |
| 4105271 | Glass in upper arm | Condition |
| 4115379 | Localized, primary osteoarthritis of the pelvic region and thigh | Condition |
| 36562855 | Poroma, NOS, of skin of other and unspecified parts of face | Condition |
| 42538151 | Osteoporosis co-occurrent and due to multiple myeloma | Condition |
| 44505541 | Bowen disease of skin of other and unspecified parts of face | Condition |
| 44782549 | Disorder of vertebral column | Condition |
| 45767042 | Pathological fracture of humerus due to osteoporosis | Condition |
| 45767043 | Pathological fracture of scapula due to osteoporosis | Condition |
| 45768892 | Osteoporosis due to cystic fibrosis | Condition |
| 45771423 | Pathological fracture of ankle due to osteoporosis | Condition |
| 45772718 | Pathological fracture of radius due to osteoporosis | Condition |
| Hypertension condition | | |
| 195556 | Hypertensive heart AND renal disease | Condition |
| 201313 | Hypertensive renal disease | Condition |
| 316866 | Hypertensive disorder | Condition |
| 317895 | Renovascular hypertension | Condition |
| 319034 | Hypertensive heart disease without congestive heart failure | Condition |
| 319826 | Secondary hypertension | Condition |
| 320128 | Essential hypertension | Condition |
| 439694 | Hypertensive heart and renal disease with both (congestive) heart failure and renal failure | Condition |
| 439695 | Hypertensive heart and renal disease with renal failure | Condition |
| 439696 | Hypertensive heart and renal disease with (congestive) heart failure | Condition |
| 442604 | Hypertensive heart disease | Condition |
| 443919 | Hypertensive renal failure | Condition |
| 444101 | Hypertensive heart failure | Condition |
| 4028806 | Primary hyperaldosteronism due to adrenal adenoma | Condition |
| 4032952 | Renal sclerosis with hypertension | Condition |
| 4108213 | Hypertension secondary to drug | Condition |
| 4110948 | Hypertension secondary to endocrine disorder | Condition |
| 4138307 | Right heart failure due to pulmonary hypertension | Condition |
| 4239507 | Hyperaldosteronism with nodular hyperplasia of adrenal cortex | Condition |
| 4243652 | Secondary dentin | Condition |
| 4289933 | Malignant hypertension | Condition |
| 4322893 | Hypertensive left ventricular hypertrophy | Condition |
| 43021748 | Hypertensive nephrosclerosis | Condition |
| 44784439 | Benign hypertensive renal disease with renal failure | Condition |
| 45768449 | Hypertensive crisis | Condition |
| Diabetes mellitus condition | | |
| 192279 | Disorder of kidney due to diabetes mellitus | Condition |
| 201254 | Type 1 diabetes mellitus | Condition |
| 201531 | Hyperosmolar coma due to type 1 diabetes mellitus | Condition |
| 201820 | Diabetes mellitus | Condition |
| 201826 | Type 2 diabetes mellitus | Condition |
| 376112 | Polyneuropathy due to diabetes mellitus | Condition |
| 376979 | Cataract due to diabetes mellitus | Condition |
| 380096 | Proliferative retinopathy due to diabetes mellitus | Condition |
| 439770 | Ketoacidosis due to type 1 diabetes mellitus | Condition |
| 443412 | Type 1 diabetes mellitus without complication | Condition |
| 443727 | Diabetic ketoacidosis | Condition |
| 443730 | Disorder of nervous system due to diabetes mellitus | Condition |
| 4008576 | Diabetes mellitus without complication | Condition |
| 4019513 | Brittle diabetes mellitus | Condition |
| 4024659 | Gestational diabetes mellitus | Condition |
| 4029423 | Hypoglycemia due to diabetes mellitus | Condition |
| 4033942 | Diabetic dermopathy | Condition |
| 4048028 | Diabetic mononeuropathy | Condition |
| 4048029 | Thoracic radiculopathy due to diabetes mellitus | Condition |
| 4065354 | Neuropathic ulcer of foot due to diabetes mellitus | Condition |
| 4082346 | Disorder of soft tissue due to diabetes mellitus | Condition |
| 4095288 | Ketoacidotic coma due to diabetes mellitus | Condition |
| 4096041 | Malnutrition-related diabetes mellitus with ketoacidosis | Condition |
| 4096042 | Malnutrition-related diabetes mellitus without complications | Condition |
| 4096670 | Malnutrition-related diabetes mellitus with renal complications | Condition |
| 4096671 | Malnutrition-related diabetes mellitus with peripheral circulatory complications | Condition |
| 4099214 | Type 1 diabetes mellitus with ulcer | Condition |
| 4099215 | Type 1 diabetes mellitus maturity onset | Condition |
| 4099216 | Multiple complications due to type 2 diabetes mellitus | Condition |
| 4099652 | Multiple complications due to malnutrition related diabetes | Condition |
| 4101887 | Mononeuritis multiplex with diabetes mellitus | Condition |
| 4105172 | Preproliferative retinopathy due to diabetes mellitus | Condition |
| 4114426 | Diabetic hand syndrome | Condition |
| 4128221 | Microalbuminuric diabetic nephropathy | Condition |
| 4131908 | Peripheral angiopathy due to diabetes mellitus | Condition |
| 4152858 | Type 1 diabetes mellitus with arthropathy | Condition |
| 4159742 | Diabetic foot ulcer | Condition |
| 4169240 | Bullosis diabeticorum | Condition |
| 4174977 | Retinopathy due to diabetes mellitus | Condition |
| 4175440 | Autonomic neuropathy due to diabetes mellitus | Condition |
| 4193704 | Type 2 diabetes mellitus without complication | Condition |
| 4195045 | Vitreous hemorrhage due to diabetes mellitus | Condition |
| 4200875 | Type 2 diabetes mellitus with peripheral angiopathy | Condition |
| 4222687 | Persistent microalbuminuria due to type 1 diabetes mellitus | Condition |
| 4224419 | Disorder of eye due to malnutrition related diabetes mellitus | Condition |
| 4225656 | Cataract due to diabetes mellitus type 1 | Condition |
| 4226798 | Hypoglycemic coma due to diabetes mellitus | Condition |
| 4228112 | Hypoglycemic coma due to type 1 diabetes mellitus | Condition |
| 4230254 | Type 2 diabetes mellitus in nonobese | Condition |
| 4242528 | Diarrhea due to diabetes mellitus | Condition |
| 4263090 | Femoral mononeuropathy due to diabetes mellitus | Condition |
| 4301699 | Neuropathy | Condition |
| 4304377 | Type 2 diabetes mellitus in obese | Condition |
| 4307319 | Symmetric proximal motor neuropathy due to diabetes mellitus | Condition |
| 4322638 | Diabetes mellitus AND insipidus with optic atrophy AND deafness | Condition |
| 4336000 | Proliferative retinopathy with neovascularization elsewhere than the optic disc due to diabetes mellitus | Condition |
| 4338901 | Traction detachment of retina due to diabetes mellitus | Condition |
| 35626762 | Cranial nerve palsy due to diabetes mellitus | Condition |
| 35626765 | Cranial nerve palsy due to type 1 diabetes mellitus | Condition |
| 36684827 | Diabetes mellitus type 2 with periodontal disease | Condition |
| 36685758 | Ketosis-prone diabetes mellitus | Condition |
| 37017429 | Gastroparesis due to type 1 diabetes mellitus | Condition |
| 37017431 | Polyneuropathy due to type 1 diabetes mellitus | Condition |
| 37017432 | Polyneuropathy due to type 2 diabetes mellitus | Condition |
| 37018196 | Prediabetes | Condition |
| 37018566 | Peripheral neuropathy due to type 1 diabetes mellitus | Condition |
| 37018765 | Gestational diabetes mellitus complicating pregnancy | Condition |
| 40480031 | Hyperglycemic crisis due to diabetes mellitus | Condition |
| 42536603 | Hyperosmolar hyperglycemic coma due to diabetes mellitus without ketoacidosis | Condition |
| 42536604 | Lactic acidosis due to diabetes mellitus | Condition |
| 42538715 | Acute complication due to diabetes mellitus | Condition |
| 43531578 | Chronic kidney disease due to type 2 diabetes mellitus | Condition |
| 43531640 | Maturity-onset diabetes of the young | Condition |
| 44793113 | Diabetes mellitus with multiple complications | Condition |
| 44808373 | Impaired glucose regulation | Condition |
| 45757535 | Microalbuminuria due to type 1 diabetes mellitus | Condition |
| 45769830 | Neuropathic arthropathy due to type 1 diabetes mellitus | Condition |
| Rheumatoid arthritis condition | | |
| 74125 | Inflammatory polyarthropathy | Condition |
| 80809 | Rheumatoid arthritis | Condition |
| 134442 | Systemic sclerosis | Condition |
| 254443 | Sjogren's syndrome | Condition |
| 255348 | Polymyalgia rheumatica | Condition |
| 256197 | Rheumatoid lung disease | Condition |
| 257628 | Systemic lupus erythematosus | Condition |
| 4027727 | Systemic sclerosis, diffuse | Condition |
| 4035427 | Rheumatoid arthritis with multisystem involvement | Condition |
| 4035611 | Seropositive rheumatoid arthritis | Condition |
| 4063581 | Drug-induced systemic lupus erythematosus | Condition |
| 4063582 | Systemic sclerosis induced by drugs and chemicals | Condition |
| 4079978 | Overlap syndrome | Condition |
| 4083556 | Seronegative rheumatoid arthritis | Condition |
| 4102493 | Polyneuropathy in rheumatoid arthritis | Condition |
| 4105026 | Myopathy due to systemic sclerosis | Condition |
| 4114439 | Rheumatoid arthritis of shoulder | Condition |
| 4115161 | Rheumatoid arthritis - hand joint | Condition |
| 4116148 | Rheumatoid arthritis of sternoclavicular joint | Condition |
| 4116149 | Rheumatoid arthritis of acromioclavicular joint | Condition |
| 4116150 | Rheumatoid arthritis of hip | Condition |
| 4116151 | Rheumatoid arthritis of knee | Condition |
| 4116441 | Rheumatoid arthritis of wrist | Condition |
| 4116444 | Rheumatoid arthritis of sacroiliac joint | Condition |
| 4116445 | Rheumatoid arthritis of ankle | Condition |
| 4134867 | Mixed collagen vascular disease | Condition |
| 4135937 | CREST syndrome | Condition |
| 4163270 | Seronegative arthritis | Condition |
| 4179877 | Autoimmune connective tissue disorder | Condition |
| 4244269 | Juvenile | Condition |
| 4265603 | Granulomatous rosacea | Condition |
| 4269880 | Rheumatoid arteritis | Condition |
| 4271003 | Rheumatoid vasculitis | Condition |
| 4344400 | Systemic lupus erythematosus with multisystem involvement | Condition |
| 37108714 | Seropositive rheumatoid arthritis of multiple joints | Condition |
| 37207809 | Seronegative rheumatoid arthritis of multiple sites | Condition |
| 40485046 | Progressive systemic sclerosis | Condition |
| 42534861 | Bilateral arthritis of glenohumeral joints | Condition |
| Renal disease condition | | |
| 192359 | Renal failure syndrome | Condition |
| 193782 | End-stage renal disease | Condition |
| 194685 | Non-obstructive reflux-associated chronic pyelonephritis | Condition |
| 197921 | Renal osteodystrophy | Condition |
| 198124 | Kidney disease | Condition |
| 198185 | Chronic renal failure | Condition |
| 252365 | Membranous glomerulonephritis | Condition |
| 433257 | Mesangiocapillary glomerulonephritis | Condition |
| 443597 | Chronic kidney disease stage 3 | Condition |
| 443601 | Chronic kidney disease stage 2 | Condition |
| 443611 | Chronic kidney disease stage 5 | Condition |
| 443612 | Chronic kidney disease stage 4 | Condition |
| 444024 | Complication of peritoneal dialysis | Condition |
| 4024519 | Uremic neuropathy | Condition |
| 4027119 | Nephrotic syndrome, diffuse endocapillary proliferative glomerulonephritis | Condition |
| 4027120 | Nephrotic syndrome, diffuse mesangiocapillary glomerulonephritis | Condition |
| 4028050 | Sclerosing glomerulonephritis | Condition |
| 4056462 | Chronic mesangial proliferative glomerulonephritis | Condition |
| 4056480 | Chronic nephritic syndrome, diffuse crescentic glomerulonephritis | Condition |
| 4058841 | Nephrotic syndrome, diffuse membranous glomerulonephritis | Condition |
| 4058842 | Nephrotic syndrome, diffuse mesangial proliferative glomerulonephritis | Condition |
| 4059463 | Chronic nephritic syndrome, diffuse endocapillary proliferative glomerulonephritis | Condition |
| 4153208 | Zonular dialysis | Condition |
| 4261202 | Uremia | Condition |
| 4272486 | Diffuse crescentic glomerulonephritis | Condition |
| 4316365 | Uremic coma | Condition |
| 4324887 | Disorder related to renal transplantation | Condition |
| 4335996 | Retinal dialysis | Condition |
| 36717583 | Dense deposit disease | Condition |
| 40317643 | Non-functioning kidney | Condition |
| 42537741 | Transplant present | Condition |
| 42539502 | Transplanted kidney present | Condition |
| 44790781 | Skin turgor moderately decreased | Condition |
| 45757748 | Hereditary diffuse mesangial proliferative glomerulonephritis | Condition |
| Mild liver disease condition | | |
| 192240 | Chronic viral hepatitis B with hepatitis D | Condition |
| 192675 | Biliary cirrhosis | Condition |
| 193256 | Alcoholic fatty liver | Condition |
| 194417 | Hepatic infarction | Condition |
| 196463 | Alcoholic cirrhosis | Condition |
| 197494 | Viral hepatitis C | Condition |
| 198964 | Chronic hepatitis C | Condition |
| 199867 | Chronic persistent hepatitis | Condition |
| 200763 | Chronic hepatitis | Condition |
| 201612 | Alcoholic liver damage | Condition |
| 439674 | Chronic viral hepatitis B without delta-agent | Condition |
| 4012113 | Chronic viral hepatitis | Condition |
| 4013083 | MCHC - low | Condition |
| 4015842 | Viral hepatitis carrier | Condition |
| 4026125 | Chronic active hepatitis | Condition |
| 4026131 | Non-alcoholic fatty liver | Condition |
| 4046123 | Secondary biliary cirrhosis | Condition |
| 4050640 | Mixed micro and macronodular cirrhosis | Condition |
| 4058695 | Toxic liver disease with fibrosis and cirrhosis of liver | Condition |
| 4059290 | Steatosis of liver | Condition |
| 4059298 | Toxic liver disease with chronic persistent hepatitis | Condition |
| 4064161 | Cirrhosis of liver | Condition |
| 4071022 | Micronodular cirrhosis | Condition |
| 4133325 | Focal nodular hyperplasia of liver | Condition |
| 4135822 | Primary biliary cholangitis | Condition |
| 4184779 | Macronodular cirrhosis | Condition |
| 4223448 | Autoimmune polyendocrinopathy | Condition |
| 4225905 | Liver cyst | Condition |
| 4232955 | Cryptogenic cirrhosis | Condition |
| 4238978 | Chronic lobular hepatitis | Condition |
| 4240725 | Peliosis hepatis | Condition |
| 4247079 | Hemangioma of liver | Condition |
| 4267417 | Hepatic fibrosis | Condition |
| 4296554 | Chronic persistent type B viral hepatitis | Condition |
| 4304584 | Portal cirrhosis | Condition |
| 4313567 | Postnecrotic cirrhosis | Condition |
| 4340383 | Alcoholic hepatitis | Condition |
| 4340385 | Alcoholic fibrosis and sclerosis of liver | Condition |
| 4340394 | Hepatic sclerosis | Condition |
| 4340948 | Hepatic fibrosis with hepatic sclerosis | Condition |
| 4340951 | Liver transplant disorder | Condition |
| 36562408 | Bowen disease of other specified parts of female genital organs | Condition |
| 37396401 | Decompensated cirrhosis of liver | Condition |
| 46271811 | Child-Pugh score class A | Condition |
| 46271812 | Child-Pugh score class B | Condition |
| 46271813 | Child-Pugh score class C | Condition |
| Heart failure condition | | |
| 316139 | Heart failure | Condition |
| 319835 | Congestive heart failure | Condition |
| 321588 | Heart disease | Condition |
| 439694 | Hypertensive heart and renal disease with both (congestive) heart failure and renal failure | Condition |
| 439696 | Hypertensive heart and renal disease with (congestive) heart failure | Condition |
| 439846 | Left heart failure | Condition |
| 442310 | Acute heart failure | Condition |
| 443580 | Systolic heart failure | Condition |
| 443587 | Diastolic heart failure | Condition |
| 444101 | Hypertensive heart failure | Condition |
| 4138307 | Right heart failure due to pulmonary hypertension | Condition |
| 4139864 | Congestive heart failure due to left ventricular systolic dysfunction | Condition |
| 4195785 | Right heart failure secondary to left heart failure | Condition |
| 4236658 | Pleural effusion due to congestive heart failure | Condition |
| 4273632 | Right ventricular failure | Condition |
| 4307356 | Cor pulmonale | Condition |
| 43020895 | Fetal left ventricular dysfunction | Condition |
| Chronic pulmonary disease condition | | |
| 252348 | Chronic pulmonary radiation disease | Condition |
| 252946 | Coal workers' pneumoconiosis | Condition |
| 254389 | Pneumoconiosis due to inorganic dust | Condition |
| 255573 | Chronic obstructive lung disease | Condition |
| 255841 | Chronic bronchitis | Condition |
| 256449 | Bronchiectasis | Condition |
| 256450 | Asbestosis | Condition |
| 257004 | Acute exacerbation of chronic obstructive airways disease | Condition |
| 257905 | Mucopurulent chronic bronchitis | Condition |
| 259044 | Pneumoconiosis | Condition |
| 261325 | Pulmonary emphysema | Condition |
| 261889 | Simple chronic bronchitis | Condition |
| 313236 | Cough variant asthma | Condition |
| 317009 | Asthma | Condition |
| 442125 | Pneumoconiosis due to silica | Condition |
| 443801 | Exercise-induced asthma | Condition |
| 444084 | Extrinsic allergic alveolitis | Condition |
| 4009890 | Pain in upper limb | Condition |
| 4028970 | Tracheobronchitis | Condition |
| 4050734 | Scar emphysema | Condition |
| 4104812 | Bronchiolectasis | Condition |
| 4110048 | Chronic bullous emphysema | Condition |
| 4110051 | Mixed asthma | Condition |
| 4110056 | Chronic obstructive pulmonary disease with acute lower respiratory infection | Condition |
| 4112826 | Mixed simple and mucopurulent chronic bronchitis | Condition |
| 4119932 | Silicotuberculosis | Condition |
| 4123254 | Factitious asthma | Condition |
| 4133623 | Bulla of lung | Condition |
| 4142782 | Anthracosilicosis | Condition |
| 4144532 | Subacute obliterative bronchiolitis due to inhalation of chemical fumes AND/OR vapors | Condition |
| 4146835 | Infantile colic | Condition |
| 4167085 | Pulmonary heart disease | Condition |
| 4191479 | Allergic asthma | Condition |
| 4193588 | Moderate chronic obstructive pulmonary disease | Condition |
| 4195892 | Chronic cor pulmonale | Condition |
| 4196712 | Mild chronic obstructive pulmonary disease | Condition |
| 4196950 | Stannosis | Condition |
| 4200851 | Chronic tracheobronchitis | Condition |
| 4209097 | Severe chronic obstructive pulmonary disease | Condition |
| 4233784 | Asthmatic bronchitis | Condition |
| 4266525 | Pulmonary siderosis | Condition |
| 4270490 | Tracheitis | Condition |
| 4278831 | Chronic diffuse emphysema due to inhalation of chemical fumes AND/OR vapors | Condition |
| 4286497 | Centriacinar emphysema | Condition |
| 4307356 | Cor pulmonale | Condition |
| 4312524 | Substance induced asthma | Condition |
| 4312602 | Laryngotracheobronchitis | Condition |
| 4321599 | Immunodeficiency with thymoma | Condition |
| 4339214 | Secondary pulmonary hypertension | Condition |
| 36562855 | Poroma, NOS, of skin of other and unspecified parts of face | Condition |
| 37116845 | Acute severe refractory exacerbation of asthma | Condition |
| 40403695 | Eisenmenger's complex | Condition |
| 40493243 | Eisenmenger's syndrome | Condition |
| 46269770 | Severe persistent allergic asthma | Condition |
| 46269776 | Mild persistent allergic asthma | Condition |
| 46269784 | Moderate persistent allergic asthma | Condition |
| Coagulation disorder condition | | |
| 432585 | Blood coagulation disorder | Condition |
| 432863 | Acquired coagulation factor deficiency | Condition |
| 432869 | Hemorrhagic disorder due to circulating anticoagulants | Condition |
| 436093 | Disseminated intravascular coagulation | Condition |
| 436483 | Postpartum coagulation defects | Condition |
| 4078700 | Lupus anticoagulant disorder | Condition |
| 4095458 | Blood coagulation pathway finding | Condition |
| 4098766 | Deficiency of coagulation factor due to liver disease | Condition |
| 4101596 | Deficiency of coagulation factor due to vitamin K deficiency | Condition |
| Anemia condition | | |
| 22281 | Sickle cell-hemoglobin SS disease | Condition |
| 28396 | Hereditary hemolytic anemia | Condition |
| 136949 | Refractory anemia with excess blasts (clinical) | Condition |
| 137829 | Aplastic anemia | Condition |
| 140681 | Constitutional aplastic anemia | Condition |
| 321080 | Hypertension complicating pregnancy, childbirth and the puerperium | Condition |
| 432295 | Pernicious anemia | Condition |
| 432452 | Anemia of prematurity | Condition |
| 432868 | Hemoglobinopathy | Condition |
| 432875 | Anemia due to chronic blood loss | Condition |
| 432881 | Pancytopenia | Condition |
| 434156 | Congenital anemia | Condition |
| 434894 | Acute posthemorrhagic anemia | Condition |
| 435789 | Megaloblastic anemia | Condition |
| 436659 | Iron deficiency anemia | Condition |
| 437247 | Anemia of chronic disease | Condition |
| 438722 | Non megaloblastic anemia associated with nutritional deficiency | Condition |
| 439777 | Anemia | Condition |
| 440977 | Megaloblastic anemia due to folate deficiency | Condition |
| 440979 | Acquired hemolytic anemia | Condition |
| 441258 | Anemia in neoplastic disease | Condition |
| 441269 | Autoimmune hemolytic anemia | Condition |
| 444238 | Anemia due to enzyme deficiency | Condition |
| 4003185 | Refractory anemia (clinical) | Condition |
| 4021911 | Megaloblastic anemia due to poor nutrition | Condition |
| 4050620 | Deficiency of glucose-6-phosphate dehydrogenase | Condition |
| 4071073 | Late anemia of newborn due to isoimmunization | Condition |
| 4098018 | Mechanical hemolytic anemia | Condition |
| 4098019 | Toxic hemolytic anemia | Condition |
| 4098131 | Myelophthisic anemia | Condition |
| 4098145 | Idiopathic aplastic anemia | Condition |
| 4099508 | Refractory anemia without sideroblasts, so stated | Condition |
| 4101001 | Chronic anemia | Condition |
| 4101582 | Aplastic anemia due to chronic disease | Condition |
| 4115393 | Hereditary nonspherocytic hemolytic anemia | Condition |
| 4120450 | Normocytic anemia due to chronic blood loss | Condition |
| 4134596 | Chronic mental disorder | Condition |
| 4144811 | Anemia due to zinc deficiency | Condition |
| 4147911 | Megaloblastic anemia due to inborn errors of metabolism | Condition |
| 4148471 | Fanconi's anemia | Condition |
| 4160887 | Cold autoimmune hemolytic anemia | Condition |
| 4175331 | Congenital hemolytic anemia | Condition |
| 4188474 | Thalassemia with other hemoglobinopathy | Condition |
| 4195171 | Normocytic hypochromic anemia | Condition |
| 4198102 | Microangiopathic hemolytic anemia | Condition |
| 4204062 | Congenital dyserythropoietic anemia | Condition |
| 4206007 | Anemia following fetal blood loss | Condition |
| 4213893 | Achlorhydric anemia | Condition |
| 4218100 | Hemolytic anemia due to drugs | Condition |
| 4224115 | Postoperative confusion | Condition |
| 4262948 | Microcytic hypochromic anemia | Condition |
| 4280354 | Nutritional anemia | Condition |
| 4318674 | Chronic idiopathic autoimmune hemolytic anemia | Condition |
| 36562855 | Poroma, NOS, of skin of other and unspecified parts of face | Condition |
| 36566583 | Gastrinoma of other specified parts of pancreas | Condition |
| 37119138 | Iron deficiency anemia due to blood loss | Condition |
| 44806268 | Refractory anaemia with multilineage dysplasia | Condition |
| Mood disorder condition | | |
| 372599 | Severe mixed bipolar I disorder without psychotic features | Condition |
| 377535 | Sleep walking disorder | Condition |
| 433455 | Sleep related bruxism | Condition |
| 434911 | Recurrent major depressive episodes, severe, with psychosis | Condition |
| 434920 | Hypochondriasis | Condition |
| 435220 | Severe recurrent major depression without psychotic features | Condition |
| 435225 | Depressed bipolar I disorder in full remission | Condition |
| 435226 | Bipolar affective disorder, current episode mixed | Condition |
| 435238 | Sleep paralysis | Condition |
| 435520 | Reactive depressive psychosis | Condition |
| 435524 | Sleep disorder | Condition |
| 436074 | Panic disorder | Condition |
| 436086 | Manic bipolar I disorder in full remission | Condition |
| 436386 | Severe depressed bipolar I disorder with psychotic features | Condition |
| 436665 | Bipolar disorder | Condition |
| 436676 | Posttraumatic stress disorder | Condition |
| 436677 | Adjustment disorder | Condition |
| 436962 | Insomnia | Condition |
| 437250 | Mild depressed bipolar I disorder | Condition |
| 437529 | Mixed bipolar I disorder in partial remission | Condition |
| 438406 | Severe major depression, single episode, with psychotic features | Condition |
| 438727 | Atypical depressive disorder | Condition |
| 439001 | Severe mixed bipolar I disorder with psychotic features | Condition |
| 439007 | REM sleep behavior disorder | Condition |
| 439013 | Insomnia disorder related to another mental disorder | Condition |
| 439251 | Bipolar affective disorder, currently depressed, in full remission | Condition |
| 439254 | Bipolar affective disorder, current episode depression | Condition |
| 439255 | Bipolar affective disorder, currently manic, in full remission | Condition |
| 439708 | Disorders of initiating and maintaining sleep | Condition |
| 439785 | Moderate mixed bipolar I disorder | Condition |
| 440078 | Bipolar affective disorder, current episode manic | Condition |
| 440079 | Mild mixed bipolar I disorder | Condition |
| 440087 | Parasomnia | Condition |
| 440092 | Non-24 hour sleep-wake cycle | Condition |
| 440383 | Depressive disorder | Condition |
| 441534 | Severe major depression, single episode, without psychotic features | Condition |
| 442570 | Severe depressed bipolar I disorder without psychotic features | Condition |
| 442600 | Manic bipolar I disorder in partial remission | Condition |
| 443414 | Chronic post-traumatic stress disorder | Condition |
| 443433 | Jet lag | Condition |
| 443454 | Cerebral infarction | Condition |
| 443544 | Organic sleep disorder | Condition |
| 443797 | Severe manic bipolar I disorder without psychotic features | Condition |
| 443906 | Mixed bipolar I disorder | Condition |
| 4009170 | Tay-Sachs disease | Condition |
| 4009184 | Dream anxiety disorder | Condition |
| 4009648 | Mixed bipolar I disorder in full remission | Condition |
| 4010963 | Cramp in lower leg associated with rest | Condition |
| 4021498 | Panic attack | Condition |
| 4025677 | Single episode of major depression in full remission | Condition |
| 4044241 | Sleep-related painful erections | Condition |
| 4047912 | Hypersomnia of non-organic origin | Condition |
| 4087475 | Not getting enough sleep | Condition |
| 4095285 | ST segment depression | Condition |
| 4098302 | Recurrent depression | Condition |
| 4102603 | Severe manic bipolar I disorder with psychotic features | Condition |
| 4102985 | Nonorganic insomnia | Condition |
| 4138617 | Psychophysiologic insomnia | Condition |
| 4141454 | Recurrent major depression in partial remission | Condition |
| 4145684 | Sleep-onset association disorder | Condition |
| 4147466 | Panic disorder with agoraphobia | Condition |
| 4150985 | Bipolar I disorder, most recent episode hypomanic | Condition |
| 4152280 | Major depressive disorder | Condition |
| 4154309 | Severe recurrent major depression with psychotic features | Condition |
| 4170260 | Nightmares | Condition |
| 4172156 | Bipolar II disorder, most recent episode hypomanic | Condition |
| 4177651 | Depressed bipolar I disorder in partial remission | Condition |
| 4186720 | Benign neonatal sleep myoclonus | Condition |
| 4189538 | Agoraphobia without history of panic disorder | Condition |
| 4196358 | Panic | Condition |
| 4211231 | Panic disorder without agoraphobia | Condition |
| 4215402 | Primary insomnia | Condition |
| 4215917 | Mild manic bipolar I disorder | Condition |
| 4232324 | Sleep terror disorder | Condition |
| 4262584 | Primary hypersomnia | Condition |
| 4263748 | Recurrent major depression in full remission | Condition |
| 4263778 | Sleep talking | Condition |
| 4280361 | Moderate depressed bipolar I disorder | Condition |
| 4282096 | Major depression, single episode | Condition |
| 4282316 | Recurrent major depression | Condition |
| 4305841 | Periodic limb movement disorder | Condition |
| 4306640 | Body dysmorphic disorder | Condition |
| 4307804 | Moderate manic bipolar I disorder | Condition |
| 4307956 | Bipolar II disorder | Condition |
| 4321835 | Agoraphobia | Condition |
| 4323418 | Major depression single episode, in partial remission | Condition |
| 4325875 | Sleep-related dissociative disorder | Condition |
| 4332998 | Hypochondriacal pain | Condition |
| 4338029 | Masked depression | Condition |
| 35610097 | Recurrent depression with current severe episode and psychotic features | Condition |
| 35610108 | Recurrent depression with current severe episode without psychotic features | Condition |
| 35610109 | Recurrent depression with current moderate episode | Condition |
| 35624748 | Bipolar I disorder, most recent episode depression | Condition |
| 37110495 | Depressive symptoms due to primary psychotic disorder | Condition |
| 40482768 | Long sleeper syndrome | Condition |
| 40483183 | Idiopathic hypersomnia associated with long sleep time | Condition |
| 40546087 | Depressed mood | Condition |
| Previous sepsis condition | | |
| 132797 | Sepsis | Condition |
| 4029281 | Sepsis syndrome | Condition |
| 4231991 | Infection due to Vibrio | Condition |
| 4327861 | Bacteremia due to Staphylococcus aureus | Condition |
| 35622880 | Early-onset neonatal sepsis | Condition |
| 37394658 | Severe sepsis | Condition |
| 37395591 | Line sepsis associated with dialysis catheter | Condition |
| 40487064 | Sepsis due to Escherichia coli | Condition |
| 40491961 | Sepsis due to Pseudomonas | Condition |
| Hypothyroidism condition | | |
| 132583 | Postablative hypothyroidism | Condition |
| 133728 | Congenital hypothyroidism | Condition |
| 137820 | Postoperative hypothyroidism | Condition |
| 138384 | Acquired hypothyroidism | Condition |
| 140673 | Hypothyroidism | Condition |
| 4030049 | Post-infectious hypothyroidism | Condition |
| 4034814 | Hypothyroid goiter, acquired | Condition |
| 4034815 | Autoimmune hypothyroidism | Condition |
| 4081998 | Congenital hypothyroidism with diffuse goiter | Condition |
| 4099205 | Irradiation hypothyroidism | Condition |
| 4130017 | Congenital hypothyroidism without goiter | Condition |
| 4130027 | Subclinical iodine deficiency hypothyroidism | Condition |
| 4135213 | Transitory neonatal hypoparathyroidism | Condition |
| 4183422 | Subclinical hypothyroidism | Condition |
| 4220368 | Secondary hypothyroidism | Condition |
| 4231548 | Hypothyroidism following radioiodine therapy | Condition |
| 4344285 | Myopathy in hypoparathyroidism | Condition |
| 36715574 | Hypoparathyroidism following procedure | Condition |
| 37016342 | Hypothyroidism caused by drug | Condition |
| 45757058 | Hypothyroidism due to thyroiditis | Condition |
| Parkinson disease condition | | |
| 374013 | Secondary parkinsonism | Condition |
| 381270 | Parkinson's disease | Condition |
| 4044061 | Drug-induced tic | Condition |
| 4046093 | Vascular parkinsonism | Condition |
| 4140090 | Parkinsonism | Condition |
| 4248716 | Neuroleptic-induced parkinsonism | Condition |
| 36716783 | Atypical Parkinsonism | Condition |
| 40485457 | Multiple system atrophy, Parkinson's variant | Condition |
| 44782422 | Dementia due to Parkinson's disease | Condition |
| 45765480 | Frontotemporal dementia with parkinsonism-17 | Condition |
| Peripheral vascular disease condition | | |
| 134380 | Erythromelalgia | Condition |
| 195834 | Atherosclerosis of renal artery | Condition |
| 198177 | Abdominal aortic aneurysm | Condition |
| 312343 | Ruptured aortic aneurysm | Condition |
| 312934 | Atherosclerosis of aorta | Condition |
| 312939 | Thromboangiitis obliterans | Condition |
| 315558 | Atherosclerosis of arteries of the extremities | Condition |
| 317305 | Stricture of artery | Condition |
| 317309 | Peripheral arterial occlusive disease | Condition |
| 317577 | Arteriosclerotic gangrene | Condition |
| 317585 | Aortic aneurysm | Condition |
| 320739 | Dissection of aorta | Condition |
| 321052 | Peripheral vascular disease | Condition |
| 321314 | Abdominal aortic aneurysm without rupture | Condition |
| 321882 | Generalized atherosclerosis | Condition |
| 433222 | Thoracoabdominal aortic aneurysm, ruptured | Condition |
| 436136 | Dissection of thoracic aorta | Condition |
| 436996 | Thoracoabdominal aortic aneurysm | Condition |
| 441051 | Thoracic aortic aneurysm without rupture | Condition |
| 441875 | Thoracic aortic aneurysm which has ruptured | Condition |
| 442774 | Intermittent claudication | Condition |
| 443210 | Disorder of vascular graft | Condition |
| 443569 | Cataract associated with infrared radiation | Condition |
| 4056440 | Open injury, subclavian artery | Condition |
| 4097439 | Acrocyanosis | Condition |
| 4099184 | Chronic ischemic colitis | Condition |
| 4114011 | Subclavian artery stenosis | Condition |
| 4121625 | Common iliac artery stenosis | Condition |
| 4128904 | Traumatic rupture of aorta | Condition |
| 4132130 | Dilatation of aorta | Condition |
| 4140448 | Aneurysm of descending aorta | Condition |
| 4148299 | Ischemic colitis | Condition |
| 4160027 | Complication of intravascular line | Condition |
| 4173464 | Rupture of aorta | Condition |
| 4188336 | Chronic ischemic enterocolitis | Condition |
| 4208072 | Common femoral artery stenosis | Condition |
| 4237654 | Ischemic enterocolitis | Condition |
| 4247790 | Chronic ischemic enteritis | Condition |
| 4248564 | Ischemic enteritis | Condition |
| 4253510 | Erythrocyanosis | Condition |
| 4256889 | Dissection of abdominal aorta | Condition |
| 4288295 | Monckeberg's medial sclerosis | Condition |
| 4292397 | Arteriospasm | Condition |
| 4311420 | Vascular insufficiency | Condition |
| 4323287 | Rupture of cord | Condition |
| 35615028 | Bilateral atherosclerosis of lower limbs with gangrene | Condition |
| 36715002 | Acropectorovertebral dysplasia | Condition |
| 36715221 | Coxopodopatellar syndrome | Condition |
| 37016882 | Dissection of thoracoabdominal aorta | Condition |
| 37209668 | Atherosclerosis of superior mesenteric artery | Condition |
| 40479625 | Atherosclerosis of artery | Condition |
| 40479758 | Pseudoaneurysm | Condition |
| 40479862 | Aneurysm of infrarenal abdominal aorta | Condition |
| 42535143 | Atherosclerosis of left iliac artery | Condition |
| Urinary tract infection condition | | |
| 81902 | Urinary tract infectious disease | Condition |
| 35610224 | Uncomplicated urinary tract infection | Condition |
| Peptic ulcer disease condition | | |
| 198798 | Dieulafoy's vascular malformation | Condition |
| 433515 | Chronic gastrojejunal ulcer with hemorrhage | Condition |
| 443883 | Acute disease | Condition |
| 4006994 | Acute peptic ulcer with hemorrhage and perforation | Condition |
| 4024842 | Recurrent duodenal ulcer | Condition |
| 4027663 | Peptic ulcer | Condition |
| 4027729 | Acute duodenal ulcer with hemorrhage | Condition |
| 4046500 | Acute peptic ulcer with hemorrhage | Condition |
| 4057053 | Acute duodenal ulcer | Condition |
| 4057953 | Acute gastric ulcer with perforation | Condition |
| 4059178 | Gastrojejunal ulcer | Condition |
| 4080599 | Gastrocolic ulcer | Condition |
| 4087594 | Acute gastric mucosal erosion | Condition |
| 4099014 | Duodenal ulcer with hemorrhage | Condition |
| 4100660 | Acute gastrointestinal hemorrhage | Condition |
| 4138962 | Acute duodenal ulcer without hemorrhage AND without perforation | Condition |
| 4146517 | Chronic peptic ulcer with perforation | Condition |
| 4147683 | Acute gastrojejunal ulcer without hemorrhage AND without perforation | Condition |
| 4150681 | Chronic gastric ulcer with perforation | Condition |
| 4163865 | Acute peptic ulcer without hemorrhage AND without perforation | Condition |
| 4169592 | Acute gastric ulcer with hemorrhage and perforation | Condition |
| 4173408 | Chronic duodenal ulcer with perforation | Condition |
| 4174044 | Chronic peptic ulcer with hemorrhage | Condition |
| 4177387 | Chronic gastrojejunal ulcer without hemorrhage AND without perforation | Condition |
| 4183009 | Jejunal ulcer | Condition |
| 4194543 | Acute peptic ulcer with perforation | Condition |
| 4195231 | Acute gastric ulcer without hemorrhage AND without perforation | Condition |
| 4198381 | Ulcer of duodenum | Condition |
| 4204479 | Deformed duodenal cap | Condition |
| 4204555 | Chronic peptic ulcer without hemorrhage AND without perforation | Condition |
| 4209746 | Duodenal ulcer without hemorrhage AND without perforation | Condition |
| 4211001 | Chronic gastric ulcer with hemorrhage | Condition |
| 4217947 | Acute gastrojejunal ulcer with hemorrhage and perforation | Condition |
| 4220691 | Coin sign | Condition |
| 4222896 | Chronic duodenal ulcer without hemorrhage AND without perforation | Condition |
| 4231580 | Acute gastric ulcer with hemorrhage | Condition |
| 4232181 | Chronic duodenal ulcer with hemorrhage | Condition |
| 4248429 | Gastric ulcer without hemorrhage AND without perforation | Condition |
| 4252406 | Perforation of uterus | Condition |
| 4265479 | Acute duodenal ulcer with perforation | Condition |
| 4271696 | Peptic ulcer with hemorrhage | Condition |
| 4274491 | Acute gastrojejunal ulcer with hemorrhage | Condition |
| 4289830 | Chronic duodenal ulcer with hemorrhage AND perforation | Condition |
| 4294973 | Chronic gastric ulcer with hemorrhage and with perforation | Condition |
| 4296611 | Chronic gastric ulcer without hemorrhage AND without perforation | Condition |
| 4321586 | Gastric ulcer with perforation | Condition |
| 4336230 | Acute duodenal ulcer with hemorrhage AND perforation | Condition |
| 4338225 | Peptic ulcer with perforation | Condition |
| 36713503 | Peptic anastomotic ulcer | Condition |
| 44784282 | Chronic antral gastritis with hemorrhage | Condition |
| 44808500 | Duodenal ulcer with obstruction | Condition |
| Cerebrovascular disease condition | | |
| 132584 | Thyroid hemorrhage and infarction | Condition |
| 141094 | Lichen | Condition |
| 194994 | Renal infarction | Condition |
| 254662 | Pulmonary infarction | Condition |
| 259862 | Malocclusion, Angle class I | Condition |
| 260759 | Malocclusion of teeth | Condition |
| 312938 | Hypertensive encephalopathy | Condition |
| 313226 | Carotid artery occlusion | Condition |
| 314667 | Nonpyogenic thrombosis of intracranial venous sinus | Condition |
| 316437 | Cerebral atherosclerosis | Condition |
| 373503 | Transient cerebral ischemia | Condition |
| 374055 | Basilar artery syndrome | Condition |
| 374371 | Stenosis of precerebral artery | Condition |
| 374384 | Cerebral ischemia | Condition |
| 375557 | Cerebral embolism | Condition |
| 376713 | Cerebral hemorrhage | Condition |
| 376714 | Vertebrobasilar artery syndrome | Condition |
| 378774 | Moyamoya disease | Condition |
| 380423 | Vertebral artery stenosis | Condition |
| 380747 | Cerebral arteritis | Condition |
| 381036 | Multiple AND bilateral precerebral artery stenosis | Condition |
| 381316 | Cerebrovascular accident | Condition |
| 381591 | Cerebrovascular disease | Condition |
| 432923 | Subarachnoid hemorrhage | Condition |
| 433149 | Primary malignant neoplasm of cerebellum | Condition |
| 433195 | Transient arterial retinal occlusion | Condition |
| 433505 | Subclavian steal syndrome | Condition |
| 433507 | Loss of teeth due to local periodontal disease | Condition |
| 434056 | Late effects of cerebrovascular disease | Condition |
| 435228 | Common variable agammaglobulinemia | Condition |
| 436430 | Nontraumatic extradural hemorrhage | Condition |
| 437306 | Transient global amnesia | Condition |
| 437308 | Basilar artery occlusion | Condition |
| 439040 | Subdural hemorrhage | Condition |
| 439296 | Vertebral artery occlusion | Condition |
| 441874 | Cerebral thrombosis | Condition |
| 442263 | Basilar artery stenosis | Condition |
| 442615 | Carotid artery stenosis | Condition |
| 443454 | Cerebral infarction | Condition |
| 443752 | Ventricular hemorrhage | Condition |
| 761790 | Nonpyogenic cerebral venous thrombosis with stroke | Condition |
| 762926 | Occlusion of internal carotid artery | Condition |
| 762934 | Cerebral infarction due to posterior cerebral artery occlusion | Condition |
| 762951 | Cerebral infarction due to anterior cerebral artery occlusion | Condition |
| 763015 | Cerebral infarction due to middle cerebral artery occlusion | Condition |
| 763094 | Anterior inferior cerebellar artery occlusion with infarction | Condition |
| 764363 | Cerebellar infarction due to occlusion of superior cerebellar artery | Condition |
| 764701 | Thrombosis of anterior cerebral artery | Condition |
| 4006976 | Chronic cerebral ischemia | Condition |
| 4009154 | Atheroma of cerebral arteries | Condition |
| 4029497 | Cerebral arterial aneurysm | Condition |
| 4031045 | Anterior choroidal artery syndrome | Condition |
| 4044413 | Stenosis of intestine | Condition |
| 4045737 | Pure motor lacunar infarction | Condition |
| 4045738 | Pure sensory lacunar infarction | Condition |
| 4045745 | Thalamic hemorrhage | Condition |
| 4045749 | Cerebral amyloid angiopathy | Condition |
| 4046237 | Infarction of optic radiation | Condition |
| 4046360 | Lacunar infarction | Condition |
| 4049072 | Ruptured aneurysm of artery | Condition |
| 4049659 | Subcortical hemorrhage | Condition |
| 4056050 | Stenosis of urinary meatus | Condition |
| 4077201 | Subarachnoid hemorrhage from basilar artery aneurysm | Condition |
| 4077958 | Subarachnoid hemorrhage from anterior communicating artery aneurysm | Condition |
| 4077959 | Subarachnoid hemorrhage from posterior communicating artery aneurysm | Condition |
| 4078016 | Aneurysm of internal carotid artery | Condition |
| 4078315 | Top of basilar syndrome | Condition |
| 4078446 | Subarachnoid hemorrhage from middle cerebral artery aneurysm | Condition |
| 4079120 | Ruptured internal carotid bifurcation aneurysm | Condition |
| 4079424 | Ruptured cerebral arteriovenous malformation | Condition |
| 4079431 | Ruptured aneurysm of middle cerebral artery | Condition |
| 4079434 | Ruptured aneurysm of posterior communicating artery | Condition |
| 4082161 | Ruptured aneurysm of basilar artery | Condition |
| 4103579 | Cortical cataract | Condition |
| 4108356 | Cerebral infarction due to embolism of cerebral arteries | Condition |
| 4108669 | Acute myocardial infarction of atrium | Condition |
| 4108952 | Subarachnoid hemorrhage from carotid siphon and bifurcation | Condition |
| 4110185 | Intracerebral hemorrhage, intraventricular | Condition |
| 4110186 | Intracerebral hemorrhage, multiple localized | Condition |
| 4110189 | Cerebral infarct due to thrombosis of precerebral arteries | Condition |
| 4110190 | Cerebral infarction due to embolism of precerebral arteries | Condition |
| 4110192 | Cerebral infarction due to thrombosis of cerebral arteries | Condition |
| 4110194 | Middle cerebral artery syndrome | Condition |
| 4110195 | Posterior cerebral artery syndrome | Condition |
| 4110197 | Occlusion and stenosis of multiple and bilateral cerebral arteries | Condition |
| 4111708 | Subarachnoid hemorrhage from vertebral artery | Condition |
| 4111710 | Brainstem stroke syndrome | Condition |
| 4111711 | Cerebellar stroke syndrome | Condition |
| 4111714 | Cerebral infarction due to cerebral venous thrombosis, non-pyogenic | Condition |
| 4111715 | Occlusion and stenosis of cerebral arteries, not resulting in cerebral infarction | Condition |
| 4111716 | Occlusion and stenosis of anterior cerebral artery | Condition |
| 4111717 | Occlusion and stenosis of posterior cerebral artery | Condition |
| 4111720 | Sequelae of subarachnoid hemorrhage | Condition |
| 4111721 | Sequelae of intracerebral hemorrhage | Condition |
| 4112018 | Basal ganglia hemorrhage | Condition |
| 4112020 | Carotid artery syndrome hemispheric | Condition |
| 4112022 | Left sided cerebral hemisphere cerebrovascular accident | Condition |
| 4112023 | Occlusion and stenosis of middle cerebral artery | Condition |
| 4112024 | Occlusion and stenosis of cerebellar arteries | Condition |
| 4112026 | Sequelae of cerebral infarction | Condition |
| 4120104 | Ruptured cerebral aneurysm | Condition |
| 4121624 | Internal carotid artery stenosis | Condition |
| 4124545 | Subacute silicosis | Condition |
| 4124843 | Common femoral aneurysm | Condition |
| 4129712 | Unruptured tubal pregnancy | Condition |
| 4131383 | Infarction of basal ganglia | Condition |
| 4138327 | Acute lacunar infarction | Condition |
| 4142739 | Thalamic infarction | Condition |
| 4144823 | Infarction of testis | Condition |
| 4145867 | Venous occlusion | Condition |
| 4145897 | Multiple lacunar infarcts | Condition |
| 4162038 | Occlusion of artery | Condition |
| 4164092 | Acute cerebrovascular insufficiency | Condition |
| 4172543 | Thalamic pain | Condition |
| 4175613 | Traumatic ulcer of oral mucosa | Condition |
| 4180871 | Spontaneous pain | Condition |
| 4185607 | Embolism | Condition |
| 4189343 | Aortic valve stenosis | Condition |
| 4201411 | Carotid cavernous fistula | Condition |
| 4210434 | Ruptured popliteal artery aneurysm | Condition |
| 4218781 | Cerebral hemisphere hemorrhage | Condition |
| 4244998 | Surgical proximal margin finding | Condition |
| 4249605 | Spasm of cerebral arteries | Condition |
| 4250018 | Cobalamin A disease | Condition |
| 4265428 | Diffuse lamellar keratitis | Condition |
| 4276505 | Aneurysm of superior mesenteric artery | Condition |
| 4299377 | Intrapontine hemorrhage | Condition |
| 4301259 | Posterior inferior cerebellar artery syndrome | Condition |
| 4304439 | Macular infarction | Condition |
| 4306943 | Epidural hemorrhage | Condition |
| 4311006 | Dural carotid cavernous fistula | Condition |
| 4311124 | Carotid artery thrombosis | Condition |
| 4311399 | Dilatation of intestine | Condition |
| 4316224 | Brain stem infarction | Condition |
| 4318373 | Senile ichthyosis | Condition |
| 4318408 | Subdural hematoma | Condition |
| 4318546 | Stenosis of ureter | Condition |
| 4319331 | Cerebellar infarction | Condition |
| 4319467 | Disorder of midbrain | Condition |
| 4326561 | Cerebellar hemorrhage | Condition |
| 4330466 | Bleeding from mouth | Condition |
| 4332246 | Aneurysm | Condition |
| 4338523 | Amaurosis fugax | Condition |
| 4341648 | Hemorrhagic infarction of intestine | Condition |
| 35610084 | Cerebral infarction due to occlusion of cerebral artery | Condition |
| 35624287 | Bilateral proximal complete obstruction of fallopian tubes | Condition |
| 36403145 | Adenocarcinoma in situ in multiple adenomatous polyposis coli of stomach, NOS | Condition |
| 36712772 | Acquired arteriovenous fistula | Condition |
| 36713608 | Entrapment of superficial branch of radial nerve | Condition |
| 36714154 | Progressive cavitating leukoencephalopathy | Condition |
| 36717315 | Stenosis of ileum | Condition |
| 36717605 | Silent cerebral infarct | Condition |
| 37016924 | Dissection of cerebral artery | Condition |
| 37116466 | Dementia due to chronic subdural hematoma | Condition |
| 37204663 | Aneurysm of internal carotid bifurcation | Condition |
| 40484120 | Small vessel cerebrovascular disease | Condition |
| 40485430 | Recurrent transient cerebral ischemic attack | Condition |
| 42535426 | Acute nontraumatic subdural hemorrhage | Condition |
| 42537642 | Aneurysm of basilar artery | Condition |
| 42538062 | Spontaneous intracranial hemorrhage | Condition |
| 42538857 | Subcortical dementia | Condition |
| 42710031 | Type I arteriovenous malformation of spinal cord | Condition |
| 42873046 | Hemorrhage in putamen | Condition |
| 43530606 | Sequela of traumatic intracranial hemorrhage | Condition |
| 43530683 | Cerebral infarction due to carotid artery occlusion | Condition |
| 43531610 | Spasticity as sequela of stroke | Condition |
| 45766077 | Dural arteriovenous fistula | Condition |
| 45766085 | Ruptured aneurysm of vertebral artery | Condition |
| 45767658 | Cerebral infarction due to thrombosis of middle cerebral artery | Condition |
| 45772786 | Cerebral infarction due to embolism of middle cerebral artery | Condition |
| 45773220 | Reversible cerebral vasoconstriction syndrome | Condition |
| 46270031 | Cerebral infarction due to occlusion of precerebral artery | Condition |
| 46272492 | Dissection of artery | Condition |
| 46273649 | Cerebral infarction due to occlusion of basilar artery | Condition |
| Lpid modifying agents | | |
| 1545992 | atorvastatin 20 MG | Drug |
| 1545994 | atorvastatin 40 MG | Drug |
| 1545998 | atorvastatin 10 MG Oral Tablet [Lipitor] | Drug |
| 1545999 | atorvastatin 20 MG Oral Tablet [Lipitor] | Drug |
| 1714307 | voriconazole 40 MG/ML Oral Suspension | Drug |
| 2055582 | ezetimibe 10 MG [ETROL] | Drug |
| 2065485 | nebivolol 5 MG / rosuvastatin 20 MG [NEBIROSTA] | Drug |
| 2065493 | nebivolol 2.5 MG / rosuvastatin 10 MG [NEBIROSTA] | Drug |
| 19033893 | ibuprofen 400 MG Oral Tablet [Ibu] | Drug |
| 19033925 | ibuprofen 800 MG Oral Tablet [Ibu] | Drug |
| 19043051 | cholestyramine resin 4000 MG Powder for Oral Suspension [Questran] | Drug |
| 19068781 | atorvastatin 80 MG Oral Tablet [Lipitor] | Drug |
| 19083557 | coal tar 20 MG/ML | Drug |
| 19102707 | fenofibrate 200 MG Oral Capsule [Lipidil Supra] | Drug |
| 19106672 | propylthiouracil 200 MG Oral Tablet | Drug |
| 19109464 | coal tar 40 MG/ML | Drug |
| 19122208 | atorvastatin 10 MG | Drug |
| 19123593 | atorvastatin 40 MG Oral Tablet [Lipitor] | Drug |
| 19125466 | 24 HR fluvastatin 80 MG Extended Release Oral Tablet [Lescol] | Drug |
| 19128151 | amlodipine 10 MG / atorvastatin 20 MG Oral Tablet [Caduet] | Drug |
| 19128153 | amlodipine 10 MG / atorvastatin 40 MG Oral Tablet [Caduet] | Drug |
| 19128163 | amlodipine 5 MG / atorvastatin 10 MG Oral Tablet [Caduet] | Drug |
| 19128165 | amlodipine 5 MG / atorvastatin 20 MG Oral Tablet [Caduet] | Drug |
| 19128167 | amlodipine 5 MG / atorvastatin 40 MG Oral Tablet [Caduet] | Drug |
| 21094411 | acipimox 250 MG [Olbetam] | Drug |
| 40164892 | metformin hydrochloride 1000 MG / sitagliptin 50 MG Oral Tablet [Janumet] | Drug |
| 40165246 | rosuvastatin calcium 10 MG Oral Tablet [Crestor] | Drug |
| 40165254 | rosuvastatin calcium 20 MG Oral Tablet [Crestor] | Drug |
| 40165262 | rosuvastatin calcium 5 MG Oral Tablet [Crestor] | Drug |
| 40165639 | pitavastatin calcium 1 MG Oral Tablet [Livalo] | Drug |
| 40165643 | pitavastatin calcium 2 MG Oral Tablet [Livalo] | Drug |
| 40165647 | pitavastatin calcium 4 MG Oral Tablet [Livalo] | Drug |
| 40171557 | amlodipine 5 MG / benazepril hydrochloride 10 MG Oral Capsule [Lotrel] | Drug |
| 40230606 | ceftaroline fosamil 600 MG Injection [Teflaro] | Drug |
| 40231907 | acetaminophen 500 MG / oxycodone hydrochloride 5 MG Oral Tablet [Roxicet] | Drug |
| 40798884 | Heptabarb | Drug |
| 40863597 | Fenofibrate / Pravastatin Oral Capsule [Pravafenix] | Drug |
| 40893556 | Vitamin E Oral Capsule [Vital 2g] | Drug |
| 41142323 | Bezafibrate 400 MG [Beza Retard Abz] | Drug |
| 41312215 | Lactulose 1 MG/MG Oral Solution [Lactuverlan] Box of 30 | Drug |
| 42708398 | ezetimibe 10 MG / simvastatin 10 MG Oral Tablet [Vytorin] | Drug |
| 42708401 | ezetimibe 10 MG / simvastatin 20 MG Oral Tablet [Vytorin] | Drug |
| 42708403 | ezetimibe 10 MG / simvastatin 40 MG Oral Tablet [Vytorin] | Drug |
| 42708405 | ezetimibe 10 MG / simvastatin 80 MG Oral Tablet [Vytorin] | Drug |
| 42799807 | menthol 145 MG/ML | Drug |
| 42935383 | Lovastatin 20 MG [LOVALORD] | Drug |
| 42936882 | Simvastatin 20 MG [SIMVALORD] | Drug |
| 42964917 | ezetimibe 10 MG / Simvastatin 10 MG [EZSTAR] | Drug |
| 42964978 | ezetimibe 10 MG / Simvastatin 20 MG [EZSTAR] | Drug |
| 42968988 | candesartan 8 MG / rosuvastatin 10 MG [TOGENON] | Drug |
| 42969027 | rosuvastatin 10 MG / telmisartan 80 MG [DUOWELL] | Drug |
| 42969036 | rosuvastatin 20 MG / telmisartan 80 MG [DUOWELL] | Drug |
| 42969045 | rosuvastatin 10 MG / telmisartan 40 MG [DUOWELL] | Drug |
| 42969054 | rosuvastatin 20 MG / telmisartan 40 MG [DUOWELL] | Drug |
| 42969087 | rosuvastatin 10 MG / valsartan 160 MG [ROVATITAN] | Drug |
| 42969090 | rosuvastatin 20 MG / valsartan 80 MG [ROVATITAN] | Drug |
| 42969096 | rosuvastatin 5 MG / valsartan 80 MG [ROVATITAN] | Drug |
| 42969099 | rosuvastatin 5 MG / valsartan 160 MG [ROVATITAN] | Drug |
| 42969224 | ezetimibe 10 MG / rosuvastatin 10 MG [CREDOUBLE] | Drug |
| 42969283 | ezetimibe 10 MG / rosuvastatin 20 MG [CREDOUBLE] | Drug |
| 42969592 | rosuvastatin 10 MG [NEUSTATIN-R] | Drug |
| 42969600 | rosuvastatin 10 MG [ROSULORD] | Drug |
| 42969784 | rosuvastatin 20 MG [NEUSTATIN-R] | Drug |
| 42969788 | rosuvastatin 20 MG [ROSULORD] | Drug |
| 42969943 | rosuvastatin 5 MG [NEUSTATIN-R] | Drug |
| 42969946 | rosuvastatin 5 MG [ROSULORD] | Drug |
| 42970624 | Pravastatin 20 MG [MEVALOTIN] | Drug |
| 42970628 | Pravastatin 10 MG [MEVALOTIN] | Drug |
| 42970632 | Pravastatin 40 MG [MEVALOTIN] | Drug |
| 42972642 | atorvastatin 20 MG / irbesartan 150 MG [ROVELITO] | Drug |
| 42972863 | atorvastatin 10 MG [LIPINON] | Drug |
| 42973052 | atorvastatin 20 MG [LIPINON] | Drug |
| 42973091 | atorvastatin 40 MG [LIPINON] | Drug |
| 43271238 | atorvastatin 20 MG / ezetimibe 10 MG Oral Tablet [Atozet] | Drug |
| 44029050 | Capreomycin 1000 MG [Capastat Im 1g] | Drug |
| 44053465 | Buchu 100 MG | Drug |
| 44180959 | Lactulose 1 MG/MG Oral Solution [Lactuverlan] Box of 60 | Drug |
| 46287499 | evolocumab 140 MG/ML Prefilled Syringe [Repatha] | Drug |
| Dugs affecting bone structure and mineralization | | |
| 1511436 | 1.17 ML romosozumab-aqqg 89.7 MG/ML Prefilled Syringe [Evenity] | Drug |
| 1524674 | zoledronic acid | Drug |
| 19047417 | cytarabine 20 MG/ML Injectable Solution [Tarabine PFS] | Drug |
| 21055959 | Ibandronate Oral Tablet [Bonviva] | Drug |
| 21164030 | Ibandronate 150 MG [Bonviva] | Drug |
| 35605708 | 5 ML iron sucrose 20 MG/ML Injection | Drug |
| 40173610 | alendronic acid 70 MG / cholecalciferol 5600 UNT Oral Tablet [Fosamax Plus D] | Drug |
| 40173613 | alendronic acid 70 MG Oral Tablet [Fosamax] | Drug |
| 40174487 | risedronate sodium 150 MG Oral Tablet [Actonel] | Drug |
| 40174495 | risedronate sodium 35 MG Oral Tablet [Actonel] | Drug |
| 40174499 | risedronate sodium 5 MG Oral Tablet [Actonel] | Drug |
| 40222447 | denosumab 60 MG/ML Injectable Solution [Prolia] | Drug |
| 40230586 | 1.7 ML denosumab 70 MG/ML Injection [Xgeva] | Drug |
| 40239274 | nortriptyline 10 MG Oral Capsule [Aventyl] | Drug |
| 42952460 | Alendronate 70 MG [FOSAQUEEN] | Drug |
| 42952529 | Alendronate Oral Solution [MASIBONE] | Drug |
| 42956212 | zoledronic acid 0.04 MG/ML [ZOMETA READY] | Drug |
| 42958477 | Alendronate 5 MG / Calcitriol 0.0005 MG [MAXMARVIL] | Drug |
| 42971443 | Cholecalciferol / Ibandronate Oral Tablet [BONVIVA PLUS] | Drug |
| 42971644 | Cholecalciferol / Risedronate Oral Tablet [RISENEX M] | Drug |
| 42971668 | Cholecalciferol / Risedronate Oral Tablet [RISENEX PLUS] | Drug |
| 44078696 | Iron 15 MG | Drug |
| Cataract procedure | | |
| 42363457 | Surgery for Cataract Or Lens-Extracapsular Or Intracapsular Extraction | Procedure |
| 42353073 | Surgery for After Cataract | Procedure |
| 42357214 | Surgery for Cataract Or Lens-Phacoemulsification | Procedure |
